# The near-zero-magnetic field alters microbial community structure and ecological functions in mangroves

**DOI:** 10.1093/ismeco/ycag098

**Published:** 2026-04-14

**Authors:** Fan-Yi Meng, Jin-Ye Li, Jinjie Zhou, Zhi-Feng Zhang, Donghua Pan, Junzhong Li, Qinghao Song, Cui-Jing Zhang, Meng Li

**Affiliations:** Archaeal Biology Centre, Synthetic Biology Research Center, Shenzhen Key Laboratory of Marine Microbiome Engineering, Key Laboratory of Marine Microbiome Engineering of Guangdong Higher Education Institutes, Institute for Advanced Study, Shenzhen University, Shenzhen 518060, Guangdong, China; Archaeal Biology Centre, Synthetic Biology Research Center, Shenzhen Key Laboratory of Marine Microbiome Engineering, Key Laboratory of Marine Microbiome Engineering of Guangdong Higher Education Institutes, Institute for Advanced Study, Shenzhen University, Shenzhen 518060, Guangdong, China; College of Life Sciences and Oceanography, Shenzhen University, Shenzhen 518060, Guangdong, China; Archaeal Biology Centre, Synthetic Biology Research Center, Shenzhen Key Laboratory of Marine Microbiome Engineering, Key Laboratory of Marine Microbiome Engineering of Guangdong Higher Education Institutes, Institute for Advanced Study, Shenzhen University, Shenzhen 518060, Guangdong, China; Archaeal Biology Centre, Synthetic Biology Research Center, Shenzhen Key Laboratory of Marine Microbiome Engineering, Key Laboratory of Marine Microbiome Engineering of Guangdong Higher Education Institutes, Institute for Advanced Study, Shenzhen University, Shenzhen 518060, Guangdong, China; School of Electrical Engineering and Automation, Harbin Institute of Technology, Harbin 150080, Heilongjiang, China; Zhengzhou Research Institute, Harbin Institute of Technology, Zhengzhou 450000, Henan, China; State Key Laboratory of Space Environment Interaction with Matters, Space Environment Simulation Research Infrastructure, Harbin Institute of Technology, Harbin 150080, Heilongjiang, China; School of Electrical Engineering and Automation, Harbin Institute of Technology, Harbin 150080, Heilongjiang, China; Archaeal Biology Centre, Synthetic Biology Research Center, Shenzhen Key Laboratory of Marine Microbiome Engineering, Key Laboratory of Marine Microbiome Engineering of Guangdong Higher Education Institutes, Institute for Advanced Study, Shenzhen University, Shenzhen 518060, Guangdong, China; Archaeal Biology Centre, Synthetic Biology Research Center, Shenzhen Key Laboratory of Marine Microbiome Engineering, Key Laboratory of Marine Microbiome Engineering of Guangdong Higher Education Institutes, Institute for Advanced Study, Shenzhen University, Shenzhen 518060, Guangdong, China

**Keywords:** near zero magnetic field, microbial community, ecological functions, mangroves

## Abstract

The geomagnetic field is a fundamental environmental factor, yet the effects of its absence-termed the near-zero-magnetic field (NZMF) on microbial communities and associated ecological functions remain poorly understood. To address this, we conducted for the first time a 30-day NZMF incubation experiment using sediments and three microorganisms isolated from mangrove ecosystems. They were exposed to two magnetic conditions: the natural geomagnetic field and a near-zero-magnetic field (<3 nT), achieved using a specially designed magnetic shielding device. We analyzed the changes in microbial community composition, co-occurrence networks, and ecological functions. Our findings showed that while the overall prokaryotic and eukaryotic microbial diversity remained unaffected, NZMF exposure significantly stimulated the growth of specific taxa such as *Geobacter* and methanogenic archaea. Microbial co-occurrence networks revealed a reduction in inter-taxa connections under NZMF. Furthermore, NZMF significantly altered the activity of key biogeochemical enzymes, reducing the content of available potassium and organic matter, and suppressing xylosidase activity, while enhancing the activity of leucine aminopeptidase and urease. Together, these results demonstrated that NZMF could reshape microbial community structure and key ecological functions, despite minimal shifts in overall diversity. These findings highlight the importance of geomagnetic fields as an underappreciated environmental factor and underscore the need for further research into the biogeochemical and biological implications of magnetic field variations.

## Introduction

The geomagnetic field, sustained by dynamic processes within Earth's core, has served as a fundamental and stable environmental factor throughout the history of life [1]. By deflecting high-energy solar and cosmic radiation, it has created and maintained conditions conducive to the emergence and evolution of biological systems [2, 3]. Its persistent presence suggests that life has evolved under its constant influence, making it a critical yet often overlooked abiotic factor in shaping biological processes [4, 5]. In particular, microorganisms demonstrate measurable sensitivity to magnetic fields. Substantial research has shown that applied magnetic fields, often significantly stronger than Earth’s natural intensity ranging between 50 and 60 microtesla (μT), can influence microbial physiology, including cellular growth rates, metabolic pathways, and enzyme activities [6]. For example, in bioengineering contexts, magnetic fields of ~30 mT have been shown to enhance methane production in anaerobic sludge by upregulating the expression of key genes like *mcrA* involved in methanogenesis [7]. These findings underscore the capacity of magnetic fields to directly modulate microbial biochemistry and genetics.

In contrast to the focus on enhanced fields, biological investigations under severely weakened or the absent geomagnetic field, termed hypomagnetic or near-zero-magnetic field (NZMF, < 3 nT), remain remarkably scarce. While limited evidence suggests that specialized magnetotactic bacteria alter magnetosome organization under near-zero fields [8], the responses of the vast majority of other microbial species are virtually unknown. Critically, it remains unclear how complex, interacting communities respond to the removal of this environmental factor. This gap limits our ability to predict the biological and ecological consequences of long-term geomagnetic weakening, a phenomenon supported by observational records [9]. Understanding how microbial communities adapt to a hypomagnetic environment is therefore an urgent and unresolved question in environmental microbiology.

Mangrove ecosystems represent an ideal and ecologically critical model in which to address this question. As tropical and subtropical intertidal ecosystems, mangroves are global carbon sinks due to their high primary productivity and substantial sedimentary organic carbon storage [10, 11]. These habitats support diverse microbial communities that drive biogeochemical cycling of carbon, nitrogen, phosphorus, and sulfur, thereby regulating ecological functions and climate change [12–15]. Ecological functions, encompassing nutrient transformation and enzymatic activities, are increasingly employed to evaluate ecological performance and stability [16–18]. Notably, the anaerobic sediments of mangroves host unique microbial taxa, such as Fe-reducing bacteria and methanogenic archaea, whose metabolisms are known to be sensitive to magnetic changes [19]. Despite the ecological importance of mangroves and the documented sensitivity of key microbial guilds to magnetic fields, it remains entirely unknown how the removal of the geomagnetic field affects the microbial community structure and ecological functions in mangroves [20].

In this study, we aimed to bridge this knowledge gap by investigating the effects of a near-zero-magnetic field on the microbial ecology of mangroves. Through controlled incubation of sediments collected from the Futian Mangrove Nature Reserve in Shenzhen, China, we tested the hypothesis that prolonged NZMF exposure would alter microbial community structure and ecological functions. Our specific objectives were to: (i) assess the response of microbial diversity and the abundance of key functional taxa under NZMF; (ii) quantify changes in ecological functions, including enzyme activities and nutrient cycling processes under NZMF; and (iii) elucidate linkages between shifts in microbial community and ecological functions. This work provides novel insights into the ecological implications of geomagnetic variability and underscores the role of magnetic fields as an understudied but potentially important factor shaping microbial-mediated ecosystem processes in a globally crucial biome.

## Materials and methods

### Sample site and incubation experiment under near-zero-magnetic field system

The sediment samples were collected from the Futian National Nature Reserve of Shenzhen, which is the only national nature reserve embedded within an urban area. As previously described [21], mangrove site (MG) and mudflat site (MF) were selected to represent our research sites. Sediment samples were carefully collected near the roots of *Kandelia candel* plants at the MG sites. We collected surface (0–20 cm) sediment samples. The study was conducted using the advanced ground-based ultra-low magnetic field environment simulation platform developed under the Space Environment Simulation Research Infrastructure at Harbin Institute of Technology. This advanced platform provided a unique experimental setting to mimic the magnetic field conditions of space for microbial culture experiments. The system is capable of precisely simulating near-zero-magnetic field conditions akin to those found in space. To evaluate the magnetic field distribution, multiple measurement probes were positioned in the core areas of both the ultra-low magnetic field incubation chamber and the control geomagnetic field incubation chamber. The ultra-low magnetic field system achieves magnetic field control precision at the nanotesla (nT) level and is calibrated daily using a fluxgate magnetometer to ensure long-term stability of the magnetic conditions. The internal magnetic field intensity of the system was stably maintained below 3 nT (Supplementary Fig. S1), thereby providing an excellent and stable near-zero-magnetic field environment for the experiments. In contrast, the magnetic field intensity in the control environment, which simulated the natural geomagnetic field, remained stable between 51 and 54 μT, consistent with that of typical Earth's surface magnetic field. Samples from the two sites were cultivated under geomagnetic (G) and near-zero-magnetic (0) fields and were collected after 14 days and 30 days incubation, resulting in a total of 36 samples (3 sampling time series × 2 magnetic treatments × 3 MG repetitions +3 sampling time series × 2 magnetic treatments × 3 MF repetitions). After the incubation, all sediment samples were subjected to subsequent analyses of microbial community structure and function.

### DNA extraction, 16S rRNA, and ITS gene sequencing and data processing

Total DNA was extracted from 0.5 g of sediment samples using a DNeasy PowerSoil kit, following the protocols provided by the manufacturer (Qiagen, Germany). Subsequently, the concentration and purity of the extracted DNA were assessed using a NanoDrop ND-2000c UV–Vis spectrophotometer (NanoDrop Technologies, USA). Prokaryotic community structures were analyzed by 16S rRNA gene amplicon sequencing with the primer pairs 515F and 806R [22]. To identify eukaryotic microbial community composition, we amplified the ITS region using the primer pairs gITS7ngs and ITS4ngs [23]. Amplicon libraries required at least 150 ng of polymerase chain reaction (PCR) products for sequencing on the NovaSeq 6000 platform (Illumina) at Guangdong Magigene Biotechnology Co., Ltd. Paired-end reads were merged using the join_paired_ends.py script from the Quantitative Insights into Microbial Ecology (QIIME) [24]. The obtained single-end sequences were processed using the QIIME 2 pipeline (version 2022.2) [25]. Briefly, sequences were denoised, dereplicated, and clustered into amplicon sequence variants (ASVs) using the DADA2 plugin. Taxonomy was assigned to ASV representatives using the *classify-sklearn* method against the SILVA v138 reference database for prokaryotes and the UNITE database for eukaryotic microorganisms [26, 27]. All samples were rarefied to a uniform depth of 50 000 sequences per sample. Alpha diversity metrics, including the richness and Simpson index, were calculated using the diversity module in QIIME 2.

### Culture conditions

*Geobacter metallireducens* GS-15 was cultured anaerobically with acetate (20 mM) as the electron donor and ferric citrate (56 mM) as the electron acceptor at 30°C [28]. *Methanococcoides* sp. FTZ1 and *Methanococcus* sp. CF, isolated from Futian mangrove sediments, were cultivated in modified DSM 141 medium at 30°C with methanol (100 mM) and H_2_/CO_2_ (80:20, v/v; 70 mM H_2_) as the substrates, respectively [19]. To investigate the effects of a near-zero-magnetic field on growth, inoculated cultures were divided into two groups: one incubated under a near-zero-magnetic field and the other under a geomagnetic field. For the methanogenic archaea (*Methanococcoides* sp. FTZ1 and *Methanococcus* sp. CF), samples were collected at 15, 24, 43, 63, 83, 103, and 123 h, with three replicates per time point, and growth was monitored by measuring the optical density at 600 nm. For *G. metallireducens* GS-15, growth was assessed by measuring ferrous iron (Fe^2+^) production using the ferrozine assay at 562 nm [29].

### Ecological functions

We measured 11 surrogates of ecological functions, including seven extracellular enzyme activities (EEA), available phosphorus, potassium, nitrogen and organic matter (Supplementary Table S1). All parameters were examined using air-dried sediment samples. The contents of available phosphorus were determined by the hydrochloric acid-ammonium fluoride extraction – molybdenum-antimony antimony colorimetric method (Bray method). The contents of available potassium were determined by cold nitric acid extraction flame photometry. The contents of available nitrogen were determined by alkaline hydrolysis diffusion method. The contents of organic matter were determined by potassium dichromate oxidation and external heating method. The seven extracellular enzymes included C-cycling, N-cycling, and P-cycling enzymes. N-acetyl-glucosaminidase (NAG) specifically catalyzes the hydrolysis of β-1, 4-glycosidic bonds in Chitin and glycoproteins. β-Xylosidase (XYS) releases xylose monomers by hydrolyzing the β-1, 4-glycosidic bond at the end of xylooligosaccharides (such as xydisaccharides). Leucine aminopeptidase (LAP) specifically hydrolyzes the N-terminal of peptide chains containing leucine residues and participates in protein catabolism. β-glucosidase (GC) releases glucose by hydrolyzing α or β-glucosidic bonds and participates in the metabolism of glycogen, cellulose, and other sugars. Acid phosphatase (ACP) is a hydrolase that catalyzes the hydrolysis of phosphate monoesters to inorganic phosphoric acid under acidic conditions. Urease (UE) mainly catalyzes the hydrolysis reaction of urea converting into ammonium ions. Cellulase (CL) is mainly responsible for the degradation of cellulose. The EEA were measured using a kit according to their instructions (Soil Enzyme Activity Assay Kit, Solarbio, China).

### Statistical analysis

Samples were classified into 12 groups based on collection site (MF [mudflat] and MG [mangrove]), magnetic categories (G [geomagnetic] and 0 [near zero-magnetic]), and sampling time (T0, D14, and D30). There were three replicates for each group. Data were expressed as mean ± SD. The significance analysis of differences in: microbial diversity metrics, relative abundances of dominant taxonomic orders/families and ecological functions were conducted using the T-test. Significance was set to *P* < .05. Prokaryotic and eukaryotic microbial community structure variation was shown through Principal Coordinate Analysis (PCoA) at genus level across groups using the *vegan* R package by Euclidean distance, and the differences between groups were calculated by Adonis test. To detect significantly different taxa between the treatments, the linear discriminant analysis (LDA) effect size (LEfSe) method was used [30]. An LDA > 2 and a significant *P* < .05 were applied to detect distinctive taxa. The random forest analysis was performed to identify the distinctive taxa using the “rfPermute” package in R. Co-occurrence analysis was based on the spearman model. The correlation coefficients among different species were calculated, and species with *P* < .05 and r > 0.8 were retained for subsequent visual analysis in Gephi [31]. The beta nearest-taxon index (βNTI) that was used to quantify the relative importance of stochastic and deterministic processes was performed with the ses.comdistnt function (abundance.weighted = TRUE) in the MicEco R package. Normalized stochasticity ratio (NST) were calculated to estimate stochasticity based on null model analysis of dissimilarity [32]. Neutral community model (NCM) were used to quantify the relative importance of stochastic processes in structuring the microbial community [33]. Pearson correlation analysis of ecological functions with prokaryotic and eukaryotic microbial taxa (top 20 relative abundance at the genus level) were conducted.

## Results and discussion

### Effects of near-zero-magnetic field on microbial diversity

**Figure 1:**
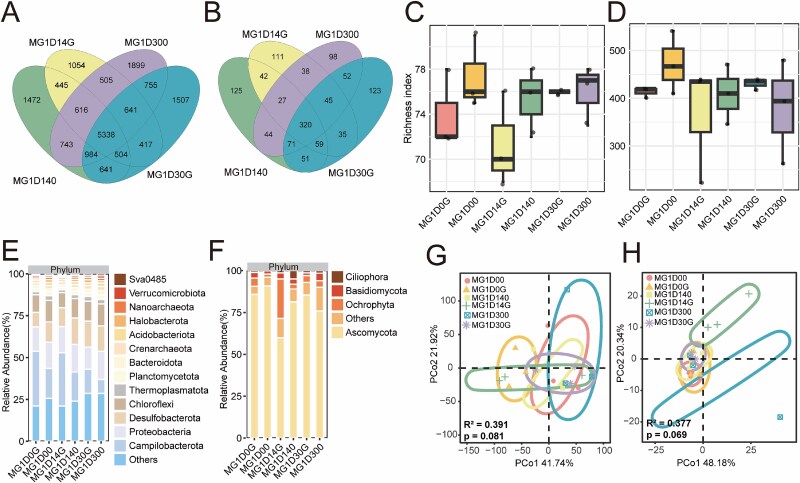
Microbial community structure in the MG group. Venn diagrams showing the numbers of shared and unique prokaryotic (A) and eukaryotic (B) OTUs. Alpha diversity (richness index) of prokaryotic (C) and eukaryotic (D) communities. Relative abundance of the dominant prokaryotic (E) and eukaryotic (F) phyla (those with an average relative abundance >1%). Beta diversity of prokaryotic (G) and eukaryotic (H) communities, presented using PCoA based on Euclidean distance (circles represent 95% confidence intervals).

We first investigated the impact of the NZMF on the diversity and composition of prokaryotic and eukaryotic microbial communities in mangrove (MG) and mudflat (MF) sediments. Venn diagram analysis revealed a substantial core microbiome shared across samples. In the MG group, 5338 prokaryotic operational taxonomic units (OTUs) were common to all samples, accounting for 30% of the total OTUs (Fig. 1A), while 320 eukaryotic OTUs were shared (26% of total; Fig. 1B). The MF group shared 6191 prokaryotic OTUs (33% of the total; Supplementary Fig. S2A) and 259 eukaryotic OTUs (22% of total; Supplementary Fig. S2B). Following the 30-day incubation under both geomagnetic and zero-magnetic conditions, near-zero-magnetic field exposure did not induce significant changes in the α-diversity of prokaryotic or eukaryotic communities, as measured by richness index, in either the MG or MF groups (Supplementary Table S2, Fig. 1C and D, Supplementary Fig. S2C and D). Analysis of the community composition identified distinct dominant phyla (Supplementary Tables S3, S4, and S5). The prokaryotic community was primarily composed of Campilobacterota (16.99%), Proteobacteria (16.43%), and Desulfobacterota (13.23%), which collectively accounted for 46.65% of the average relative abundance (Fig. 1E and Supplementary Fig. S2E). In contrast, the eukaryotic community was dominated by Ascomycota (78.70%), Basidiomycota (7.16%), and Ochrophyta (6.13%), together comprising 91.99% of the average relative abundance (Fig. 1F and Supplementary Fig. S2F). Principal Coordinate Analysis (PCoA) based on Euclidean distances further reinforced this structural stability, showing no significant separation in either prokaryotic or eukaryotic community composition between the geomagnetic and zero-magnetic treatments (Fig. 1G and H, Supplementary Fig. S2G and H). This resilience in taxonomic diversity and overall community structure is ecologically significant, suggesting that mangrove microbial communities possess a considerable buffer capacity, allowing them to maintain structural integrity in the face of a weakened geomagnetic field [6]. This stability could be attributed to functional redundancy within mangrove ecosystems [12], where the loss or enhancement of one taxon is compensated for by others with similar metabolic capabilities, thereby preserving the overall community profile [18]. Furthermore, it indicates that the near-zero-magnetic field may not act as a strong selective pressure on broad taxonomic lineages over this experimental timeframe.

### Indicative microorganisms under near-zero-magnetic field

**Figure 2:**
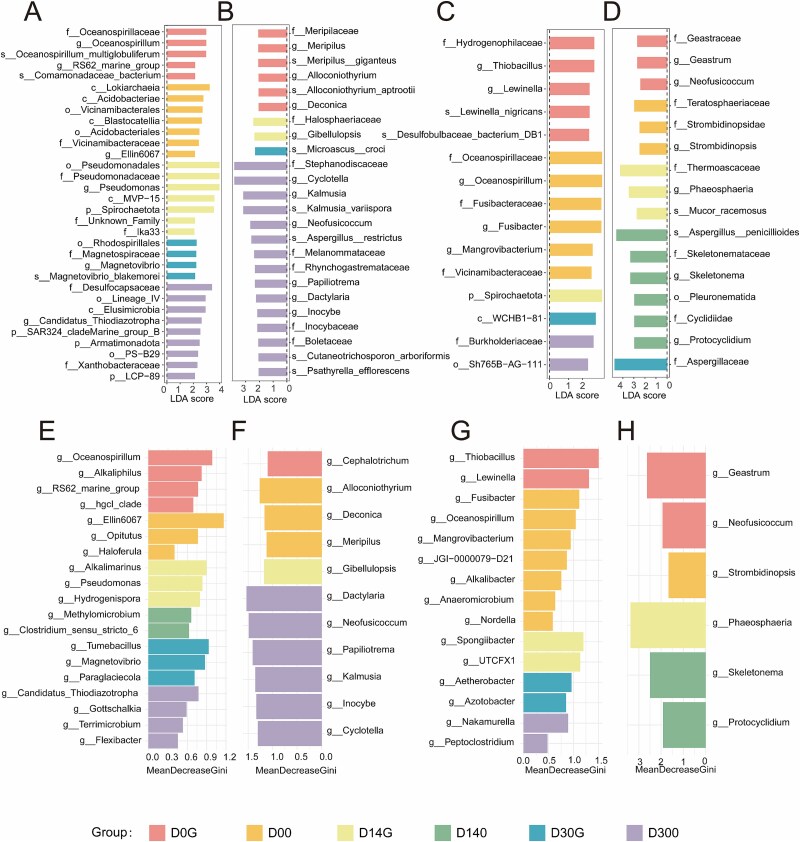
Differential microbial taxa identified among different treatments. LDA scores (LDA ≥ 2) from LEfSe analysis of prokaryotic communities in the MG (A) and MF (C) groups, and for eukaryotic communities in the MG (B) and MF (D) groups. Random forest analysis at the genus level identifies the most important discriminative taxa for prokaryotes in the MG (E) and MF (G) groups, and for fungi in the MG (F) and MF (H) groups.

To identify specific prokaryotic and eukaryotic microorganisms responding to the NZMF exposure, we performed LEfSe and random forest analysis. LEfSe results revealed that several taxa were significantly enriched under the geomagnetic field (GMF) and NZMF (Supplementary Table S6). In the MG group, the prokaryotic families *Desulfocapsaceae* and *Pseudomonadaceae* (LDA scores >3) were distinctive in the NZMF treatment, whereas the genus *Magnetovibrio* was significantly enriched under the GMF (Fig. 2A). For eukaryotic microbial communities in MG, significant taxa (LDA >3) under NZMF included *Stephanodiscaceae*, *Cyclotella,* and *Kalmusia* (Fig. 2B). In the MF group, the prokaryotic taxa *Burkholderiaceae* and Sh765B − AG − 111 (LDA >2) were identified as significantly associated with the NZMF (Fig. 2C). The eukaryotic microbial genera *Aspergillus* and *Skeletonema* (LDA >3) were indicative of NZMF exposure (Fig. 2D). Random forest analysis, used to determine the most important discriminative genera, corroborated, and extended these findings (Supplementary Table S7). In MG, prokaryotic taxa such as *Thiodiazotropha*, *Gottschalkia,* and *Terrimicrobium* (MeanDecreaseGini >0.4) showed relatively high importance under NZMF, while the genus *Magnetovibrio* was identified as a key discriminator under GMF conditions (Fig. 2E). For eukaryotic microbes, *Neofusicoccum*, *Papiliotrema*, *Kalmusia*, *Inocybe*, *Cyclotella,* and *Dactylaria* (MeanDecreaseGini >1.0) were among the most impactful taxa (Fig. 2F). In MF, important prokaryotic genera included *Nakamurella* and *Peptoclostridium* (MeanDecreaseGini >0.4) (Fig. 2G), while *Skeletonema*, and *Protocyclidium* (MeanDecreaseGini >1) were key eukaryotic indicators under NZMF (Fig. 2H).

To further evaluate the physiological response to magnetic conditions, we compared the growth kinetics and metabolic activities of specific microorganisms. NZMF significantly promoted the growth of *Geobacter* GS-15 and enhanced its Fe(III) reduction efficiency (Supplementary Fig. S3A). The growth of two methanogenic archaea—*Methanococcoides* sp. FTZ1 (methylotrophic) and *Methanococcus* sp. CF (hydrogenotrophic)—was also significantly affected. Although no notable differences were observed during the lag phase (before 24 h), the zero-magnetic group exhibited significantly higher optical density (OD) values during the exponential growth phase (after 24 h) compared to the geomagnetic group (Supplementary Fig. S3B and C). The 16S rRNA gene amplicon sequencing of the culture systems further supported these pure-culture observations. In MG, the relative abundance of *Geobacter* was higher in the zero-magnetic treatment group than in the geomagnetic control group (Supplementary Fig. S3D).

The consistent identification by both LEfSe and random forest analyses strongly indicates that certain microbial taxa exhibit sensitivity to reduction of the geomagnetic field. More importantly, the convergence between culture-independent community analyses and pure-culture growth experiments provides compelling evidence that NZMF can directly stimulate specific microbial functional groups. The significant enrichment of taxa such as *Desulfocapsaceae*, *Pseudomonadaceae*, and *Burkholderiaceae* under NZMF conditions suggests that microorganisms involved in distinct biogeochemical processes—specifically sulfur cycling, organohalide respiration, and iron reduction—may possess specialized sensory or metabolic mechanisms that are influenced by magnetic fields [34]. The members of *Geobacter* are renowned for their ability to perform extracellular electron transfer (EET) to insoluble terminal electron acceptors like ferric iron (Fe(III)). This process is facilitated by specialized molecular components, including multi-heme *c*-type cytochromes and electroactive type IV pili [35]. The enhanced relative abundance of *Geobacter* in sediments under NZMF, coupled with the stimulated Fe(III) reduction observed in pure cultures, strongly suggests that NZMF conditions may enhance EET processes. The consistent growth pattern observed—with differences manifesting primarily during exponential phase rather than lag phase—suggests that magnetic fields likely affect cellular machinery involved in replication and energy generation rather than initial adaptation mechanisms [36, 37].

### Effects of near-zero-magnetic field on microbial co-occurrence patterns

**Figure 3:**
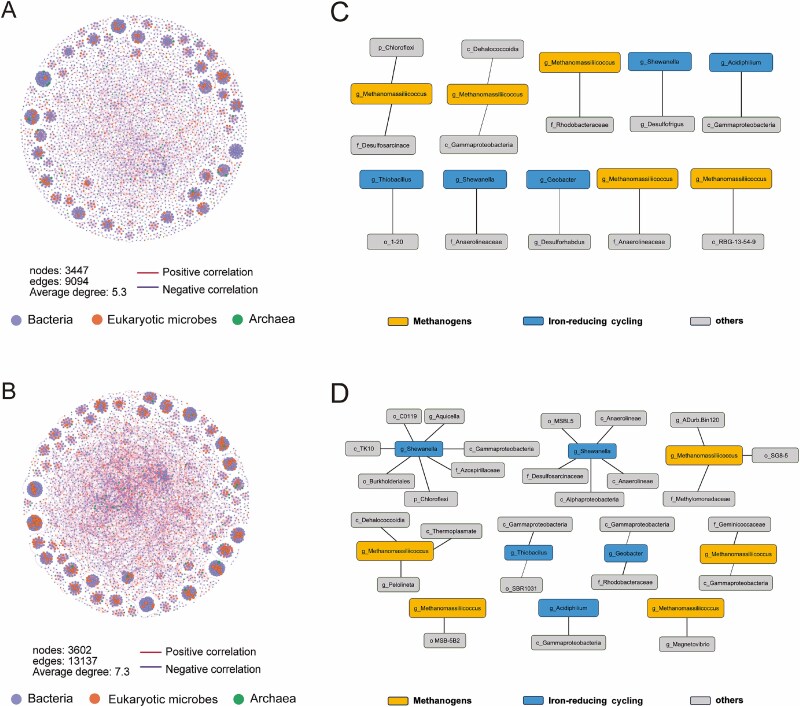
Microbial interaction networks in the MG group under NZMF and GMF conditions. Taxonomic association networks at the phylum level under NZMF (A) and GMF (B). Interaction networks involved in methanogens (yellow) or iron-cycling bacteria (blue) and other microbial taxa under NZMF (C) and GMF (D).

To investigate how NZMF conditions influence the inter-taxa associations among bacteria, archaea, and eukaryotes, we constructed phylum-level co-occurrence networks and computed key topological metrics, including nodes, edges, and average degrees for both MG and MF sediments. In MG, prokaryotic nodes primarily belonged to *Chloroflexi*, *Proteobacteria*, *Desulfobacterota*, *Thermoplasmatota,* and *Campilobacterota*. The integrated prokaryotic–eukaryotic network under NZMF contained 3447 nodes, 9094 edges, and an average degree of 5.3 (Fig. 3A), compared to 3602 nodes, 13 137 edges, and an average degree of 7.3 under geomagnetic conditions (Fig. 3B). Moreover, under NZMF conditions, there is no direct edge between methanogenic archaea (mainly *g_ Methanomasilliicoccus*) and iron-cycling bacteria (*g_ Shewanella, g_ Geobacter, g_ Thiobacillus*); they reside in different small modules, and each shows a markedly reduced number of links to surrounding bacterial taxa, presenting an effectively isolated state with limited potential for metabolic interactions (Fig. 3C). In contrast, under GMF conditions, although there remains no direct edge between methanogens and iron-reducers, both groups are embedded in denser, more complex local networks, forming many more positive and negative correlations with a variety of bacterial and archaeal taxa (Fig. 3D). This topology suggests that when the geomagnetic field is present, methanogenic archaea and iron-reducing bacteria become indirectly coupled through shared cooperative or competitive partners—the potential for metabolic connections is much greater—whereas under NZMF this indirect coupling is significantly weakened. In the MF group, the combined prokaryotic—eukaryotic network under near-zero-magnetic field consisted of 3934 nodes, 12 610 edges, and an average degree of 6.4 (Supplementary Fig. S4A), while under geomagnetic field, the values were 3871, 11 570, and 6.0, respectively (Supplementary Fig. S4B).

Our network analyses reveal that zero-magnetic conditions significantly alter microbial co-occurrence patterns. The NZMF led to a clear reduction in network complexity and connectivity, as evidenced by decreases in the number of edges and average degree in integrated networks. This suggests that the geomagnetic field may play a previously unrecognized role in maintaining or facilitating microbial interactions, possibly by influencing cellular processes such as motility, intercellular signaling, or metabolic coordination [38, 39]. Complex microbial networks are often associated with functional stability and efficient resource cycling [40]. The simplified network structure under NZMF implies a potential disruption of synergistic relationships or niche partitioning among microorganisms, which could ultimately affect community stability and functional resilience. The reduction in edges and average degree may indicate weakened interactions—such as cross-feeding, co-metabolism, or signal exchange—among key taxonomic groups. This structural simplification could render the community more vulnerable to environmental perturbations and reduce its functional redundancy.

### Effects of near-zero-magnetic field on microbial community assembly processes

**Figure 4:**
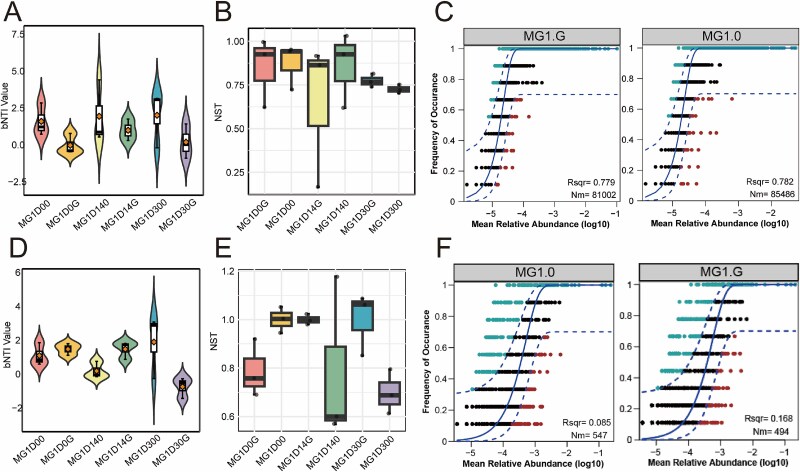
Microbial community assembly processes in the MG group. Analysis of the βNTI for prokaryotes (A) and eukaryotic microbes (D). The NST model of prokaryotes (B) and eukaryotic microbes (E). Neutral model analysis of prokaryotes (C) and eukaryotic microbes (F).

To elucidate the ecological mechanisms governing microbial community assembly under different magnetic conditions, we applied a null model-based approach using the βNTI and NST analyses. These methods quantify phylogenetic turnover between samples to evaluate the relative contributions of deterministic (e.g. environmental filtering, biotic interactions) versus stochastic processes (e.g. ecological drift, random dispersal) in community assembly. In MG, stochastic processes predominantly governed both prokaryotic and eukaryotic community assembly. For prokaryotes, the average βNTI was −0.41, with 65% of sample pairs exhibiting significant phylogenetic turnover (|βNTI| < 2) (Fig. 4A). This pattern was further supported by an NST value of 0.788, indicating that stochastic processes accounted for ~78.8% of prokaryotic community variation (Fig. 4B). The neutral community model effectively explained species distributions, with R^2^ values of 0.782 and 0.779 under zero-magnetic and geomagnetic conditions, respectively (Fig. 4C). For eukaryotic communities in MG, stochastic dominance was even more pronounced. The average βNTI was −0.56, with 71% of sample pairs showing |βNTI| < 2 (Fig. 4D). The NST value reached 0.878, reflecting an 87.8% contribution of stochastic processes to eukaryotic community assembly (Fig. 4E). However, the neutral model exhibited limited explanatory power for eukaryotes (R^2^ = 0.168 under both conditions), suggesting additional non-random factors influence eukaryotic distributions (Fig. 4F). In MF, similar assembly patterns emerged and stochastic processes also predominantly governed both prokaryotic and eukaryotic community assembly (Supplementary Fig. S5).

### Effects of near-zero-magnetic field on ecological functions

**Figure 5:**
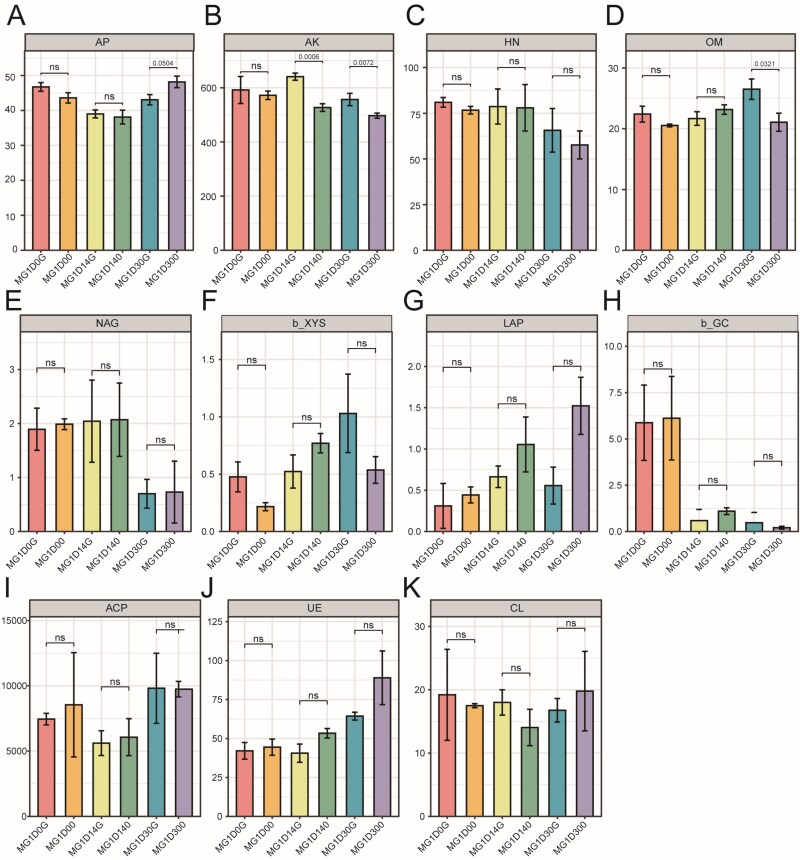
Comparison of ecological functions in the MG group under NZMF and GMF conditions. Statistically significant differences between the two magnetic conditions are indicated by asterisks (^*^) for *P* < .05, (^**^) for *P* < .01, and “ns” denotes non-significant differences. The significance was determined by one-way ANOVA followed by Duncan’s post-hoc test.

To evaluate the effects of NZMF on microbial ecological functions, this study comprehensively analyzed sediment nutrients, extracellular enzyme activities (EEAs), and their associations with microbial taxa in both MG and MF sediments (Supplementary Table S8 and S9). In MG sediments, NZMF exposure led to significant decreases in available potassium (AK) and organic matter (OM) content (Fig. 5B and D), while leucine aminopeptidase (LAP) activity was significantly enhanced (Fig. 5G), and the carbon-degrading enzyme β-glucosidase (GC) showed a decreasing trend (Fig. 5H). This indicates that NZMF simultaneously promoted organic nitrogen degradation and suppressed certain carbon cycling processes. In MF sediments, AK and OM were also significantly reduced under NZMF (Supplementary Fig. S6B and D). Furthermore, β-xylosidase (β-XYS) activity was significantly inhibited (Supplementary Fig. S6F), whereas LAP and urease (UE) activities were significantly increased (Supplementary Fig. S6G and J), further corroborating the differential regulatory effect of NZMF on carbon- and nitrogen-cycling related enzyme activities. Comprehensive analysis suggests that NZMF may enhance nitrogen and phosphorus mineralization to compensate for nutrient limitations while inhibiting certain carbon degradation pathways, thereby influencing the balance of elemental cycling and microbial energy utilization strategies in sedimentary environments.

**Figure 6:**
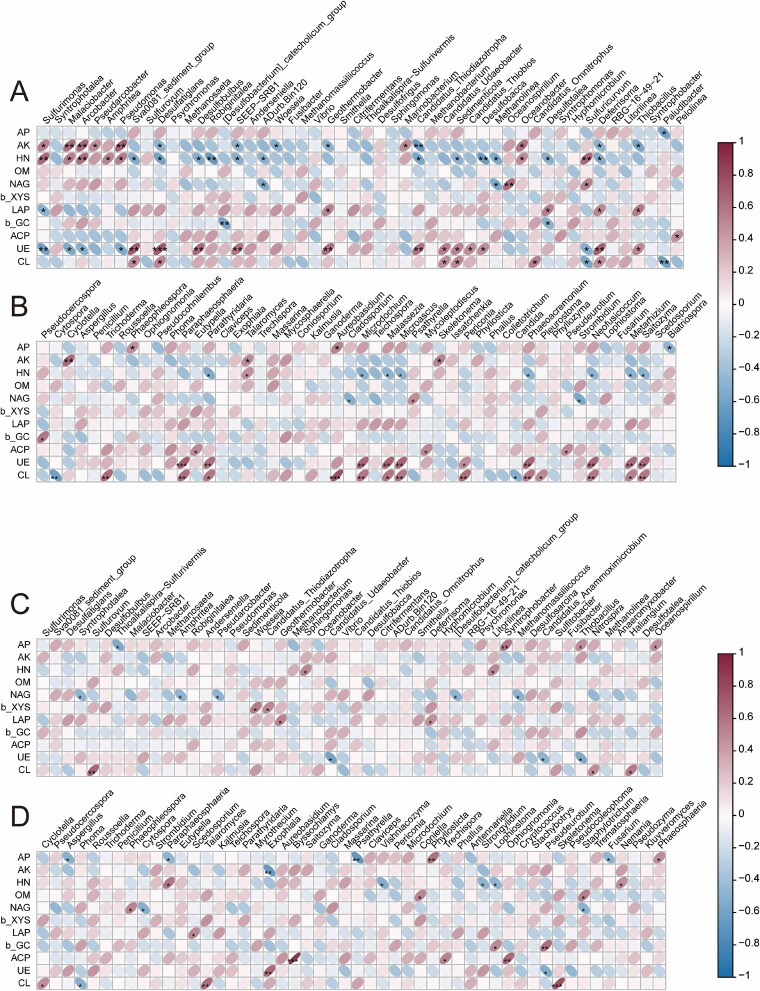
Pearson correlation analysis between ecological function parameters and dominant microbial taxa. Heatmaps showing Pearson correlation coefficients between measured parameters and the top 20 most abundant prokaryotic genera in the MG (A) and MF (C) groups. Heatmaps showing Pearson correlation coefficients between measured parameters and the top 20 most abundant eukaryotic genera in the MG (B) and MF (D) groups. Statistically significances are indicated by asterisks (^*^) for *P* < .05*,* (^**^) *P* < .01, (^***^) *P* < .001.

Further correlation analysis between microbial communities and environmental factors revealed that in MG sediments, various prokaryotic genera involved in sulfur cycling and methane metabolism, including *Sulfurimonas*, *Sulfurovum, Desulfosarcina*, *Desulfobulbus*, and *Methanomassilliicoccus*, showed significant positive or negative correlations with physicochemical factors (AP, AK, HN, OM) and multiple EEAs (NAG, β-XYS, LAP, β-GC, ACP, UE, CL) (Fig. 6A). Among the eukaryotic microbes such as *Pseudocercospora*, *Aspergillus*, *Trichoderma*, and *Cladosporium* were also significantly correlated with carbon- and nitrogen-hydrolyzing enzyme activities (Fig. 6B), suggesting their deep involvement in organic matter decomposition and nutrient remineralization. In MF sediments, although some sulfate-reducing bacteria, methanogenic archaea, and saprotrophic eukaryotic microbes still showed significant correlations with nutrient indicators and enzyme activities (Fig. 6C and D), the specific combinations of correlated microbial taxa and their response patterns differed markedly from those in MG, reflecting spatial heterogeneity in microbe-environment interactions across sedimentary environments.

Our integrated analysis of microbial functional potential, environmental parameters, and extracellular enzyme activities demonstrates that NZMF significantly influence microbial ecological functions through multiple interconnected mechanisms. The consistent reduction in available potassium and organic matter under NZMF conditions across both sediment types suggests that magnetic field reduction may accelerate microbial nutrient mineralization processes or alter nutrient cycling pathways [41]. This phenomenon could be attributed to NZMF-induced changes in microbial metabolic strategies, particularly the observed enhancements in extracellular enzyme activities involved in nitrogen and phosphorus acquisition. The elevated activities of LAP and UE under ZMF conditions indicate a fundamental shift in microbial nutrient acquisition strategies toward enhanced nitrogen and organic phosphorus mineralization [42]. This enzymatic response likely represents a compensatory mechanism for nutrient acquisition under altered environmental conditions, possibly driven by the reduced availability of key nutrients observed in NZMF treatments. The suppression of XYS activity in mudflat sediments further suggests that NZMF may differentially affect carbon and nitrogen cycling enzymes, potentially disrupting the stoichiometric balance of nutrient cycling processes. The significant enrichment of methane metabolism-related functions, despite their low abundance, highlights a potentially important role of ZMF in regulating greenhouse gas dynamics. This finding is particularly relevant given the increasing concern about climate feedback loops involving methane release from natural ecosystems.

## Conclusion

This study under a zero-magnetic environment suggests, for the first time, that the geomagnetic field could be a relevant factor for microbial communities, and thus moves beyond its well-documented role in animal magnetoreception (Supplementary Fig. S7). The observed decoupling of taxonomic structure from ecosystem function—where community composition remained stable but key processes like nutrient cycling, extracellular electron transfer, and microbial interaction networks were significantly disrupted—represents a paradigm shift in environmental microbiology. It suggests that microbial communities can maintain structural homeostasis while undergoing profound functional reprogramming in response to subtle environmental cues. However, this study also highlights significant uncertainties. The mechanistic link between the magnetic field and cellular processes in microorganisms remains a “black box.” A key limitation is the relatively short-term nature of the incubation, which may not capture long-term evolutionary adaptations or cascading effects on ecosystem stability. Future research must prioritize elucidating the molecular sensors and transduction pathways that enable microbial magnetoreception. Employing multi-omics approaches (transcriptomics, proteomics) under controlled magnetic conditions will be essential to identify the genetic basis of this phenomenon. Furthermore, long-term in situ experiments and investigations across a wider range of ecosystems are needed to determine the full scale of biogeochemical consequences. This work opens a new frontier at the intersection of geophysics and microbiology, with ramifications for understanding ecosystem function in environments with naturally low or varying magnetic fields, such as the deep subsurface or potential extraterrestrial habitats.

## Supplementary Material

SupplementaryFIGS_ycag098

SupplementaryTables_ycag098

Supplementary_ycag098

## Data Availability

The sequencing raw data generated during the current study are available in the NCBI Sequence Read Archive (SRA) under the accession number PRJNA1390668. The data are publicly available.
