# Supplementary material for: The near-zero-magnetic field alters microbial community structure and ecological functions in mangroves
Source: ISME Commun. 2026 Apr 14;6(1):ycag098. doi: 10.1093/ismeco/ycag098 (PMC13174272; doi:10.1093/ismeco/ycag098)
Supplement: SupplementaryFIGS_ycag098 [file supplementaryfigs_ycag098.pdf]

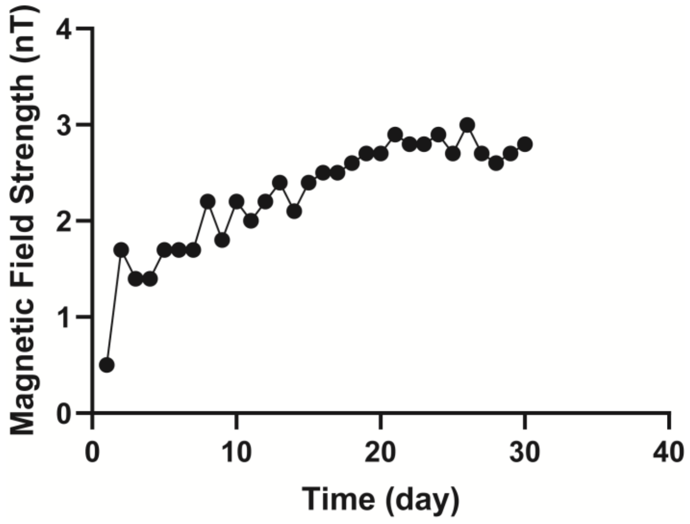

Magnetic Field Strength (nT)
0
1
2
3
4
0
10
20
30
40
Time (day)

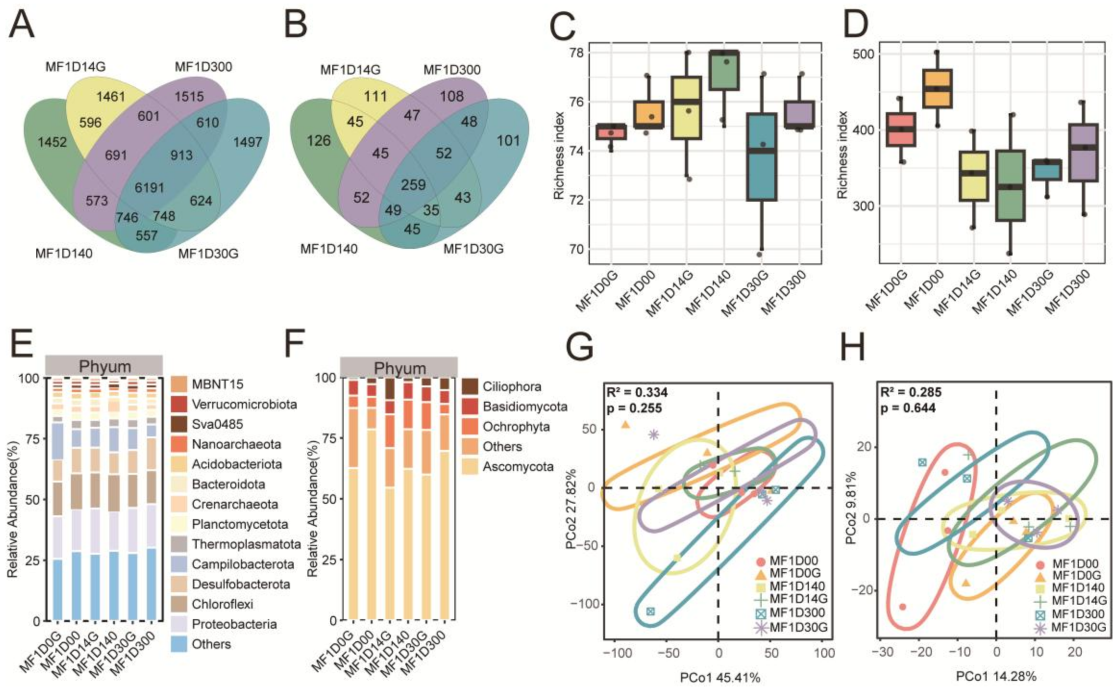
A
MF1D14G
MF1D300
MF1D140
MF1D30G
1461
1515
596
601
610
1452
691
913
1497
6191
573
624
746
748
557
B
MF1D14G
MF1D300
MF1D140
MF1D30G
111
108
45
47
48
126
45
52
101
259
52
49
35
43
45
C
Richness index
MF1D0G
MF1D00
MF1D14G
MF1D140
MF1D30G
MF1D300
D
Richness index
MF1D0G
MF1D00
MF1D14G
MF1D140
MF1D30G
MF1D300
E
Phyum
Relative Abundance(%)
MBNT15
Verrucomicrobiota
Sva0485
Nanoarchaeota
Acidobacteriota
Bacteroidota
Crenarchaeota
Planctomycetota
Thermoplasmatota
Campilobacterota
Desulfobacterota
Chloroflexi
Proteobacteria
Others
MF1D0G
MF1D00
MF1D14G
MF1D140
MF1D30G
MF1D300
F
Phyum
Relative Abundance(%)
Ciliophora
Basidiomycota
Ochrophyta
Others
Ascomycota
MF1D0G
MF1D00
MF1D14G
MF1D140
MF1D30G
MF1D300
G
R² = 0.334
p = 0.255
PCo2 27.82%
PCo1 45.41%
MF1D00
MF1D0G
MF1D140
MF1D14G
MF1D300
MF1D30G
H
R² = 0.285
p = 0.644
PCo2 9.81%
PCo1 14.28%
MF1D00
MF1D0G
MF1D140
MF1D14G
MF1D300
MF1D30G

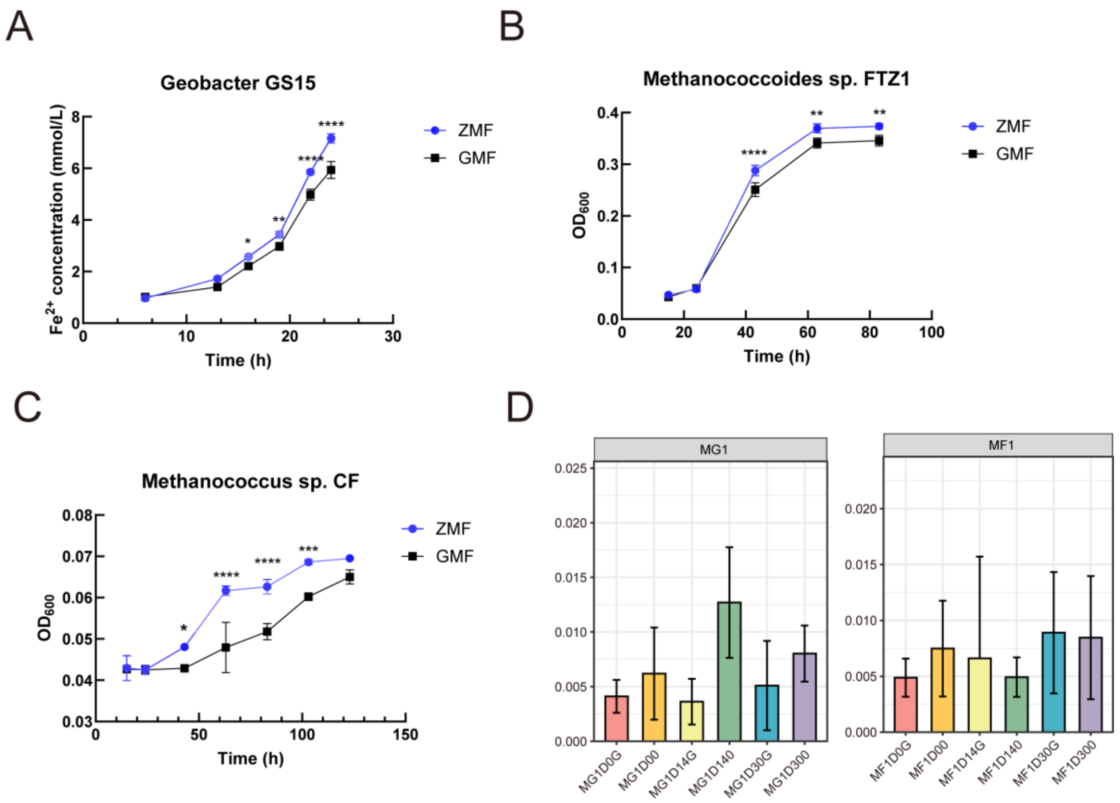
A
Geobacter GS15
Fe2+ concentration (mmol/L)
Time (h)
ZMF
GMF
B
Methanococcoides sp. FTZ1
OD600
Time (h)
ZMF
GMF
C
Methanococcus sp. CF
OD600
Time (h)
ZMF
GMF
D
MG1
MF1
MG1D0G
MG1D00
MG1D14G
MG1D140
MG1D30G
MG1D300
MF1D0G
MF1D00
MF1D14G
MF1D140
MF1D30G
MF1D300

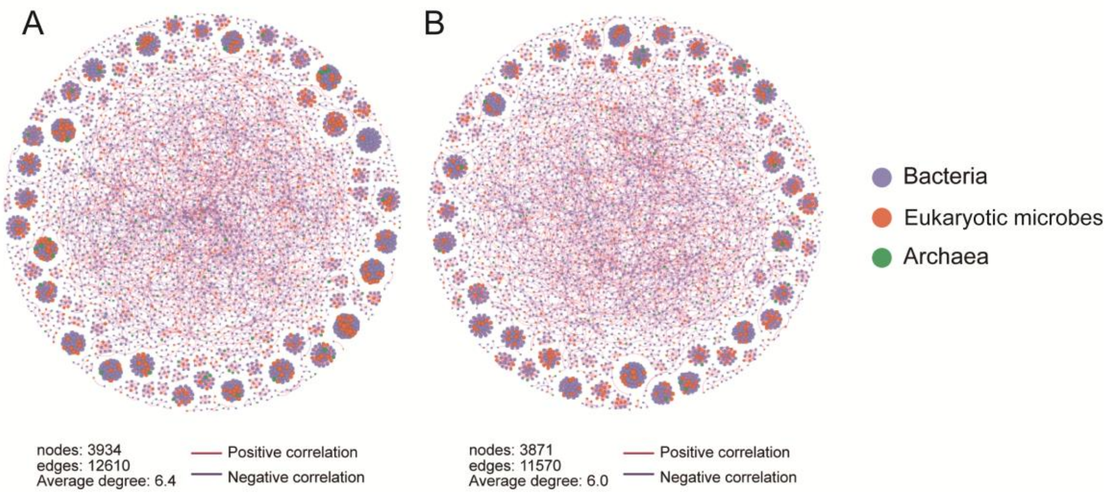
A
B
Bacteria
Eukaryotic microbes
Archaea
nodes: 3934
edges: 12610
Average degree: 6.4
Positive correlation
Negative correlation
nodes: 3871
edges: 11570
Average degree: 6.0
Positive correlation
Negative correlation

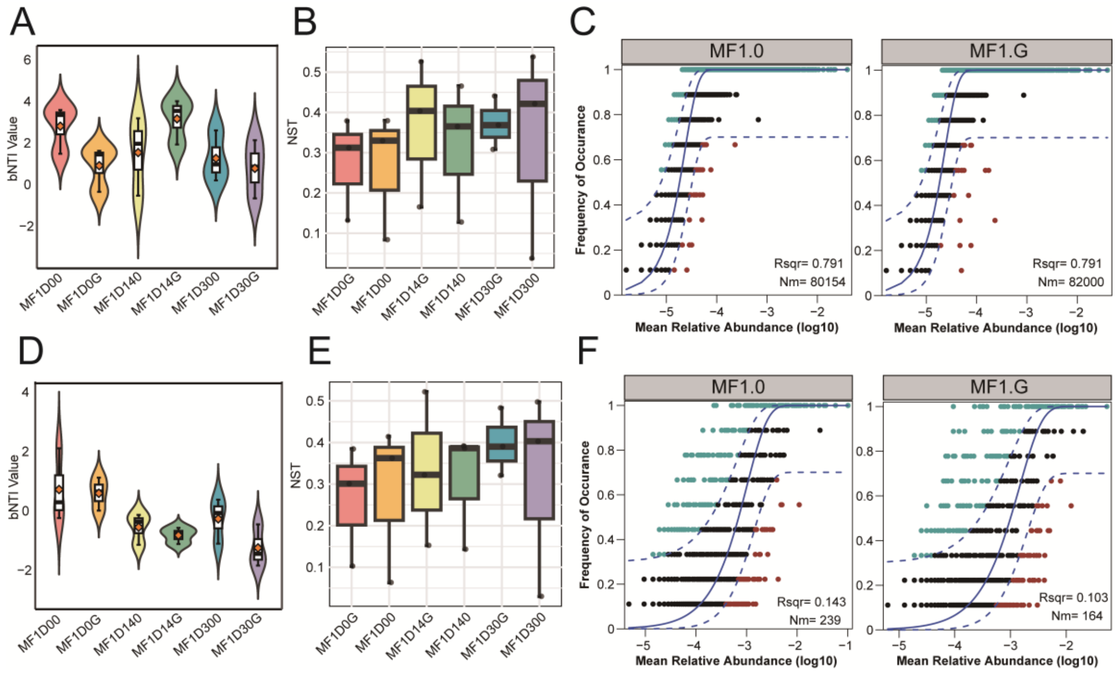

A
B
C
D
E
F
bNTI Value
NST
Frequency of Occurance
Mean Relative Abundance (log10)
MF1D00
MF1D0G
MF1D140
MF1D14G
MF1D300
MF1D30G
MF1.0
MF1.G
Rsqr= 0.791
Nm= 80154
Rsqr= 0.791
Nm= 82000
Rsqr= 0.143
Nm= 239
Rsqr= 0.103
Nm= 164

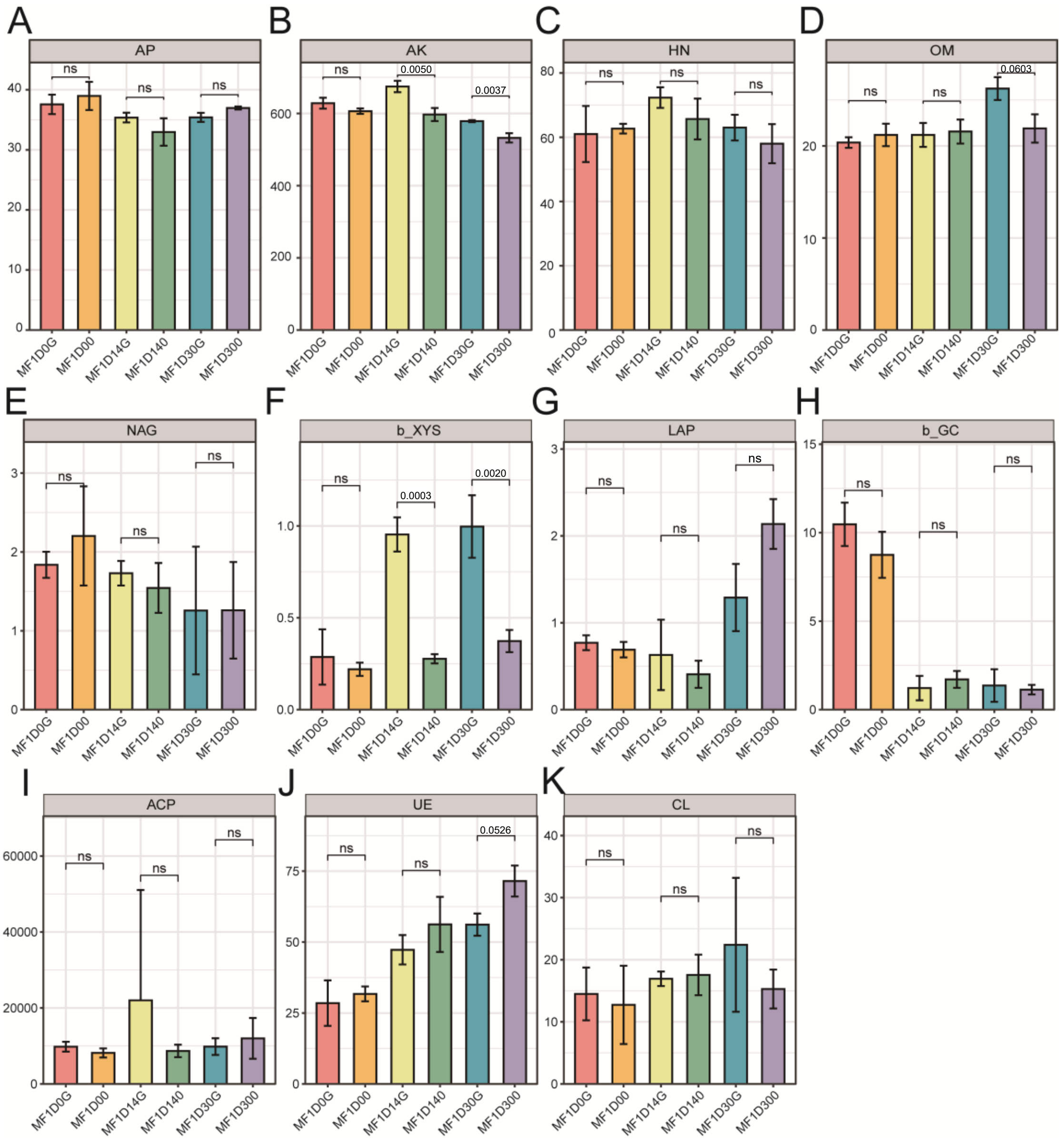
A
AP
ns
ns
ns
B
AK
ns
0.0050
0.0037
C
HN
ns
ns
ns
D
OM
ns
ns
0.0603
E
NAG
ns
ns
ns
F
b_XYS
ns
0.0003
0.0020
G
LAP
ns
ns
ns
H
b_GC
ns
ns
ns
I
ACP
ns
ns
ns
J
UE
ns
ns
0.0526
K
CL
ns
ns
ns
MF1D0G
MF1D00
MF1D14G
MF1D140
MF1D30G
MF1D300

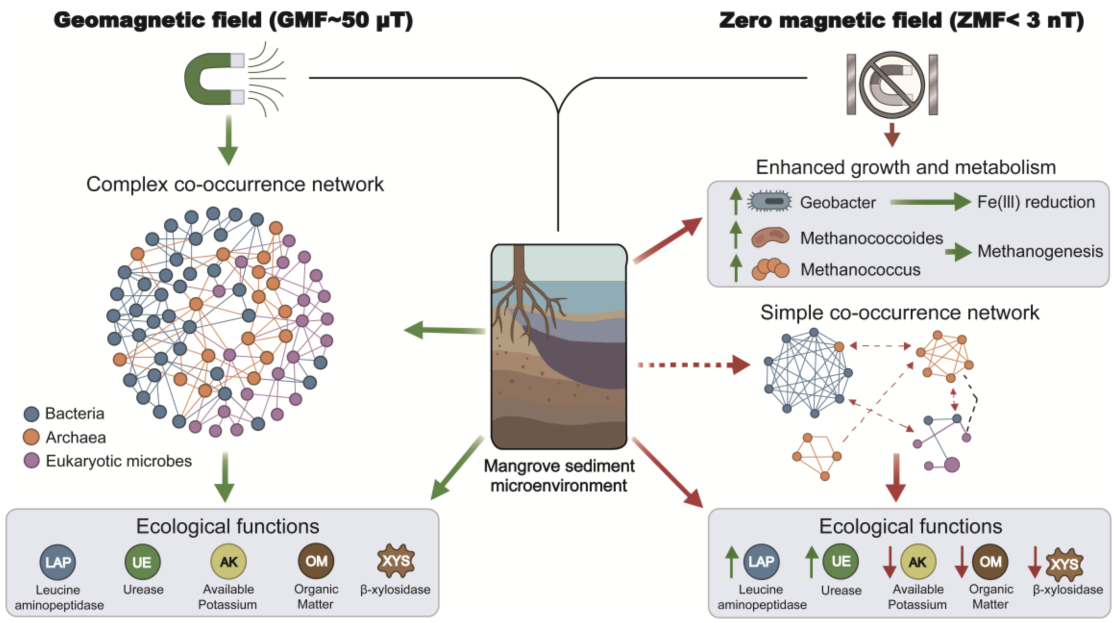
Geomagnetic field (GMF~50 μT)
Zero magnetic field (ZMF< 3 nT)
Complex co-occurrence network
Bacteria
Archaea
Eukaryotic microbes
Enhanced growth and metabolism
Geobacter
Fe(III) reduction
Methanococcoides
Methanococcus
Methanogenesis
Simple co-occurrence network
Mangrove sediment microenvironment
Ecological functions
LAP
Leucine aminopeptidase
UE
Urease
AK
Available Potassium
OM
Organic Matter
XYS
β-xylosidase
Ecological functions
LAP
Leucine aminopeptidase
UE
Urease
AK
Available Potassium
OM
Organic Matter
XYS
β-xylosidase
