# Supplementary material for: The near-zero-magnetic field alters microbial community structure and ecological functions in mangroves
Source: ISME Commun. 2026 Apr 14;6(1):ycag098. doi: 10.1093/ismeco/ycag098 (PMC13174272; doi:10.1093/ismeco/ycag098)
Supplement: Supplementary_ycag098 [file supplementary_ycag098.docx]

**Supplementary Figure 1.** Time-course monitoring of the residual magnetic field intensity under the 30-day near-zero-magnetic field condition.

**Supplementary Figure 2.** Microbial community structure in the MF group. Venn diagrams showing the numbers of shared and unique prokaryotic (a) and eukaryotic (b) OTUs. Alpha diversity (richness index) of prokaryotic (c) and eukaryotic (d) communities. Relative abundance of the dominant prokaryotic (e) and eukaryotic (f) phyla (those with an average relative abundance > 1%). Beta diversity of prokaryotic (g) and eukaryotic (h) communities, presented using PCoA based on Euclidean distance (circles represent 95% confidence intervals).

**Supplementary Figure 3.** Growth of cultured microorganisms and their relative abundance in sediment samples. Growth curves of *Geobacter metallireducens* GS-15 (a), *Methanococcoides* sp. FTZ1 (b), and *Methanococcus* sp. CF (c) under NZMF and GMF conditions. Statistically significant differences at specific time points are indicated by asterisks (*) for *P* < 0.05*,* (**) *P* < 0.01, (***) *P* < 0.001. Relative abundance of *Geobacter* based on 16S rRNA gene amplicon sequencing in sediment samples from the MG and MF groups under NZMF and GMF conditions (d).

**Supplementary Figure 4.** Microbial interaction networks in the MF group under NZMF and GMF conditions. Taxonomic association networks at the phylum level under NZMF (a) and GMF (b).

**Supplementary Figure 5.** Microbial community assembly processes in the MF group. Analysis of the βNTI for prokaryotes (a) and eukaryotic microbes (d). The NST model of prokaryotes (b) and eukaryotic microbes (e). Neutral model analysis of prokaryotes (c) and eukaryotic microbes (f).

**Supplementary Figure 6.** Comparison of ecological functions in the MF group under NZMF and GMF conditions. Statistically significant differences between the two magnetic conditions are indicated by asterisks (*) for *P* < 0.05, (**) for *P* < 0.01, and “ns” denotes non‑significant differences. The significance was determined by one‑way ANOVA followed by Duncan’s post‑hoc test.

**Supplementary Figure 7.** Graphical abstract. The NZMF alters microbial community structure and ecological functions in mangroves.
